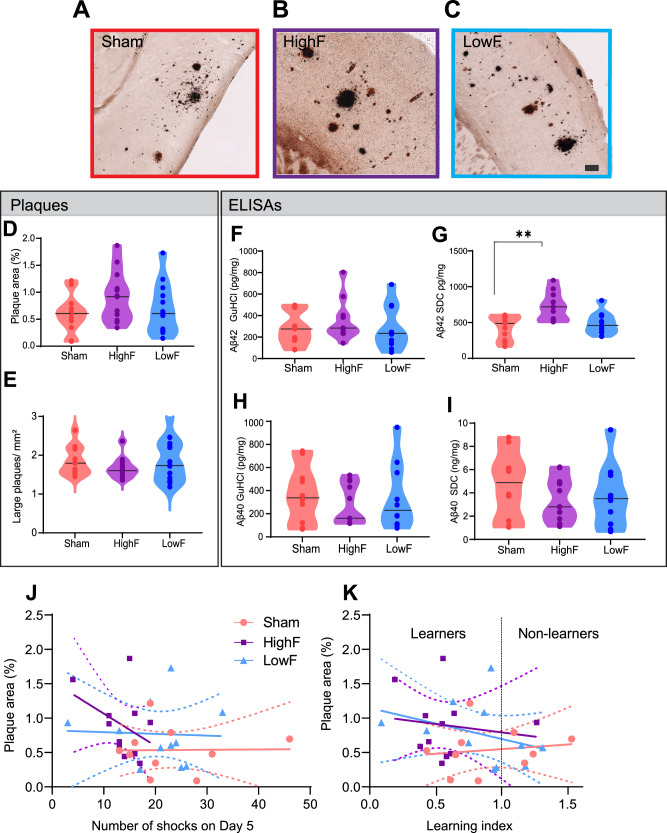# Correction: Scanning ultrasound-mediated memory and functional improvements do not require amyloid-β reduction

**DOI:** 10.1038/s41380-024-02616-3

**Published:** 2024-06-04

**Authors:** Gerhard Leinenga, Xuan Vinh To, Liviu-Gabriel Bodea, Jumana Yousef, Gina Richter-Stretton, Tishila Palliyaguru, Antony Chicoteau, Laura Dagley, Fatima Nasrallah, Jürgen Götz

**Affiliations:** 1 Clem Jones Centre for Ageing Dementia Research, Queensland Brain Institute, The University of Queensland, Brisbane, QLD Australia; 2 Queensland Brain Institute, The University of Queensland, Brisbane, QLD Australia; 3 Centre for Advanced Imaging, The University of Queensland, Brisbane, QLD Australia; 4 Proteomics Facility, Advanced Technology and Biology Division, The Walter and Eliza Hall Institute of Medical Research, Melbourne, VIC Australia; 5 Department of Medical Biology, The University of Melbourne, Parkville, VIC 3052 Australia

**Keywords:** Neuroscience, Diseases

Correction to: *Molecular Psychiatry* 10.1038/s41380-024-02509-5, published online 18 March 2024

Figure 2 in the original version of this article has been replaced. The figure 2 should have appeared as shown below.