# Supplementary material for: Scanning ultrasound-mediated memory and functional improvements do not require amyloid-β reduction
Source: Mol Psychiatry. 2024 Mar 18;29(8):2408–23. doi: 10.1038/s41380-024-02509-5 (PMC11412907; doi:10.1038/s41380-024-02509-5)
Supplement: Supplementary file 1 — Supplementary figures [file 41380_2024_2509_MOESM1_ESM.pdf]

Supplementary information

Scanning ultrasound-mediated memory and functional improvements do not require amyloid-β reduction

Gerhard Leinenga[1], Xuan Vinh To[2,3], Liviu-Gabriel Bodea[1], Jumana Yousef[4,5], Gina Richter-Stretton[1], Tishila Palliyaguru[1], Antony Chicoteau[1], Laura Dagley[4,5], Fatima Nasrallah[2,3], and Jürgen Götz[1,*]

[1]Clem Jones Centre for Aging Dementia Research, Queensland Brain Institute, The University of Queensland, Brisbane, QLD, Australia; [2]Queensland Brain Institute, The University of Queensland, Brisbane, QLD, Australia; [3]Centre for Advanced Imaging, The University of Queensland, Brisbane, QLD, Australia [4]Proteomics Facility, The Walter and Eliza Hall Institute of Medical Research, Melbourne, VIC, Australia; [5]Department of Medical Biology, The University of Melbourne, Parkville, VIC, 3052, Australia

*Correspondence: j.goetz@uq.edu.au

Supplementary figures

Supplementary Figure 1.

Treatment-frequency independent changes in the proteome. (A) Principal component analysis (PCA) reveals the presence of a sham cluster independent of the ultrasound treated samples, that shows higher similarity, with HighF and LowF treatments clustering together. (B) Volcano plot of differentially expressed proteins, displaying fold change (logFC, $\log_2$ scale) and *P* values ($-\log_{10}$ scale) between HighF treated versus sham treated controls (B) and LowF treated versus sham treated controls (C). Analysis of the proteomic data identifies expression patterns (left), top significant biological processes (middle) and expression networks (right) induced by the HighF and LowF ultrasound treatment. Cluster 3 is defined by a similar pattern of increase in the proteome triggered by ultrasound application. The cluster is represented by exocytosis and axon-associated processes. (B) Cluster 4 is characterized by a decrease in proteins following ultrasound application, that is independent of the treatment regime. The processes associated with this cluster are related to axonal biology and vesicle localization.

Supplementary Figure 2.

Diffusion MRI metrics correlated with performance in the APA test. (A) The neurite dispersion index (NDI) is positively correlated with the number of shocks received in the APA test in ultrasound treated mice. (B) The orientation dispersion index (ODI) was positively correlated with the number of shocks received in the APA test in ultrasound treated mice, with a higher ODI in the corpus collosum being correlated with a better performance in the APA test. (The heat map indicates voxels with $p < 0.05$ correlation. Indicative coronal and mid-sagittal slices are displayed.)

Supplementary Figure 3.

Levels of synaptic tau are reduced in ultrasound treated mice and this correlates with behavioral improvements in the ultrasound treated APP23 mice. (A) Tau was detected in the post-synaptic density (PSD) fraction of synaptosomes isolated from the hippocampus using the antibody tau-5, by normalizing to REVERT total protein stain. (B) Quantification of synaptic tau and the results of a one-way ANOVA followed by Holm-Sidak multiple comparison test. (C) Levels of phospho-tau detected with the anti-pSer404 tau antibody were low in the PSD fraction of hippocampal synaptosomes and unaltered by ultrasound. (D) Quantification of synaptic tau phosphorylated at serine 404 and the results of a one-way ANOVA followed by Holm-Sidak multiple comparison test. (E) The PSD fraction is enriched in PSD95 as expected, compared to the hippocampal homogenate. (F) Synaptic tau levels plotted against performance in the APA test for the different groups. (G) Linear regression of data points from all groups combined (WT, HighF, LowF and sham) reveals a significant correlation between synaptic tau levels and performance in the APA test as shown by linear regression analysis. (H) Levels of tau in cortical homogenates were the same across treatment groups. (I) Levels of phosphorylated tau at Ser404 were unaltered in cortical extracts after ultrasound treatment.

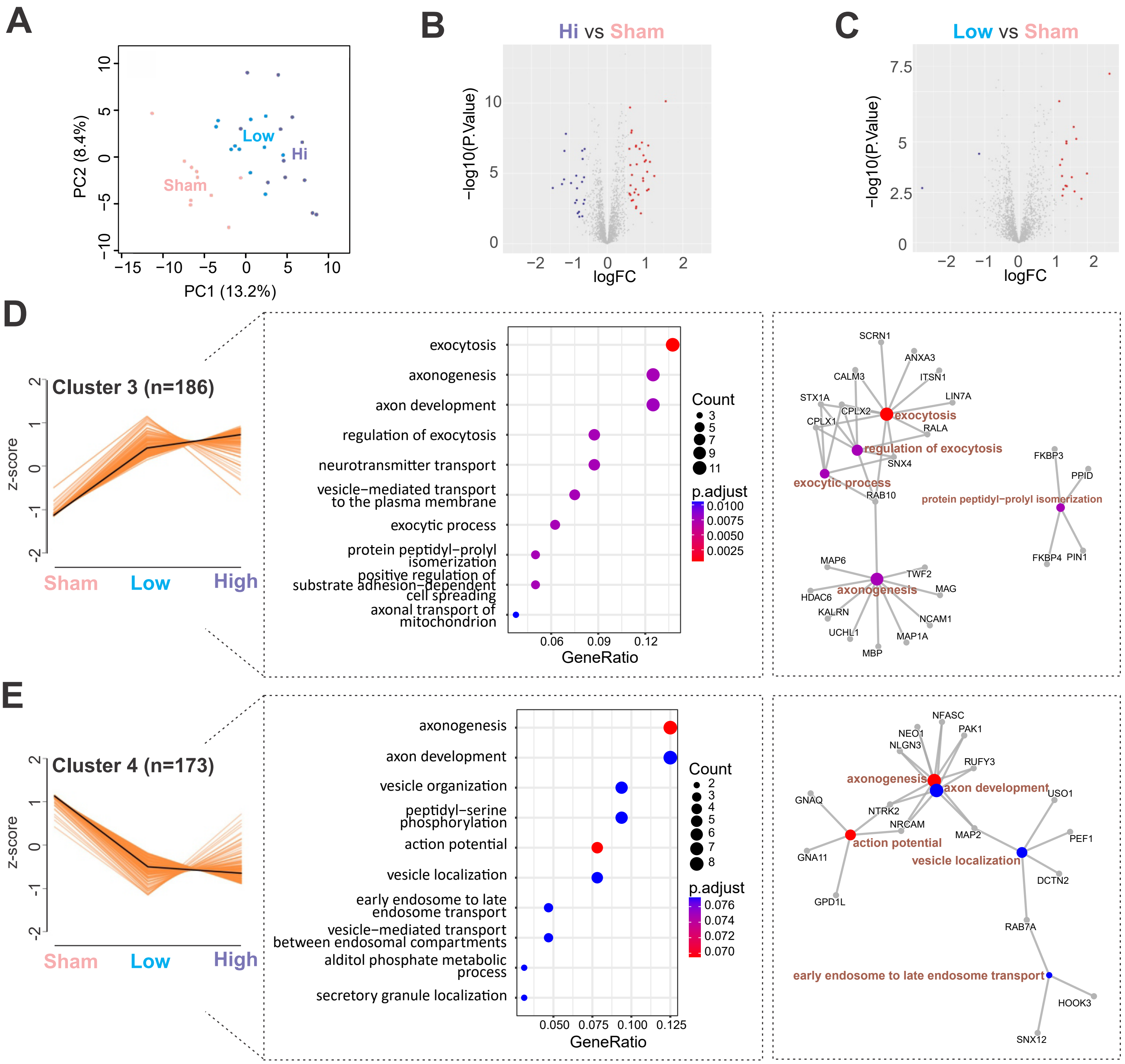
A
PC2 (8.4%)
PC1 (13.2%)
Low
Hi
Sham
B
Hi vs Sham
−log10(P.Value)
logFC
C
Low vs Sham
−log10(P.Value)
logFC
D
Cluster 3 (n=186)
z-score
Sham
Low
High
exocytosis
axonogenesis
axon development
regulation of exocytosis
neurotransmitter transport
vesicle−mediated transport to the plasma membrane
exocytic process
protein peptidyl−prolyl isomerization
positive regulation of substrate adhesion−dependent cell spreading
axonal transport of mitochondrion
GeneRatio
Count
p.adjust
SCRN1
ANXA3
CALM3
ITSN1
LIN7A
STX1A
CPLX2
CPLX1
RALA
regulation of exocytosis
SNX4
exocytic process
RAB10
FKBP3
PPID
protein peptidyl−prolyl isomerization
FKBP4
PIN1
MAP6
TWF2
HDAC6
MAG
KALRN
NCAM1
UCHL1
MAP1A
MBP
E
Cluster 4 (n=173)
z-score
Sham
Low
High
axonogenesis
axon development
vesicle organization
peptidyl−serine phosphorylation
action potential
vesicle localization
early endosome to late endosome transport
vesicle−mediated transport between endosomal compartments
alditol phosphate metabolic process
secretory granule localization
GeneRatio
Count
p.adjust
NFASC
NEO1
PAK1
NLGN3
RUFY3
GNAQ
USO1
NTRK2
NRCAM
MAP2
PEF1
GNA11
DCTN2
GPD1L
RAB7A
HOOK3
SNX12

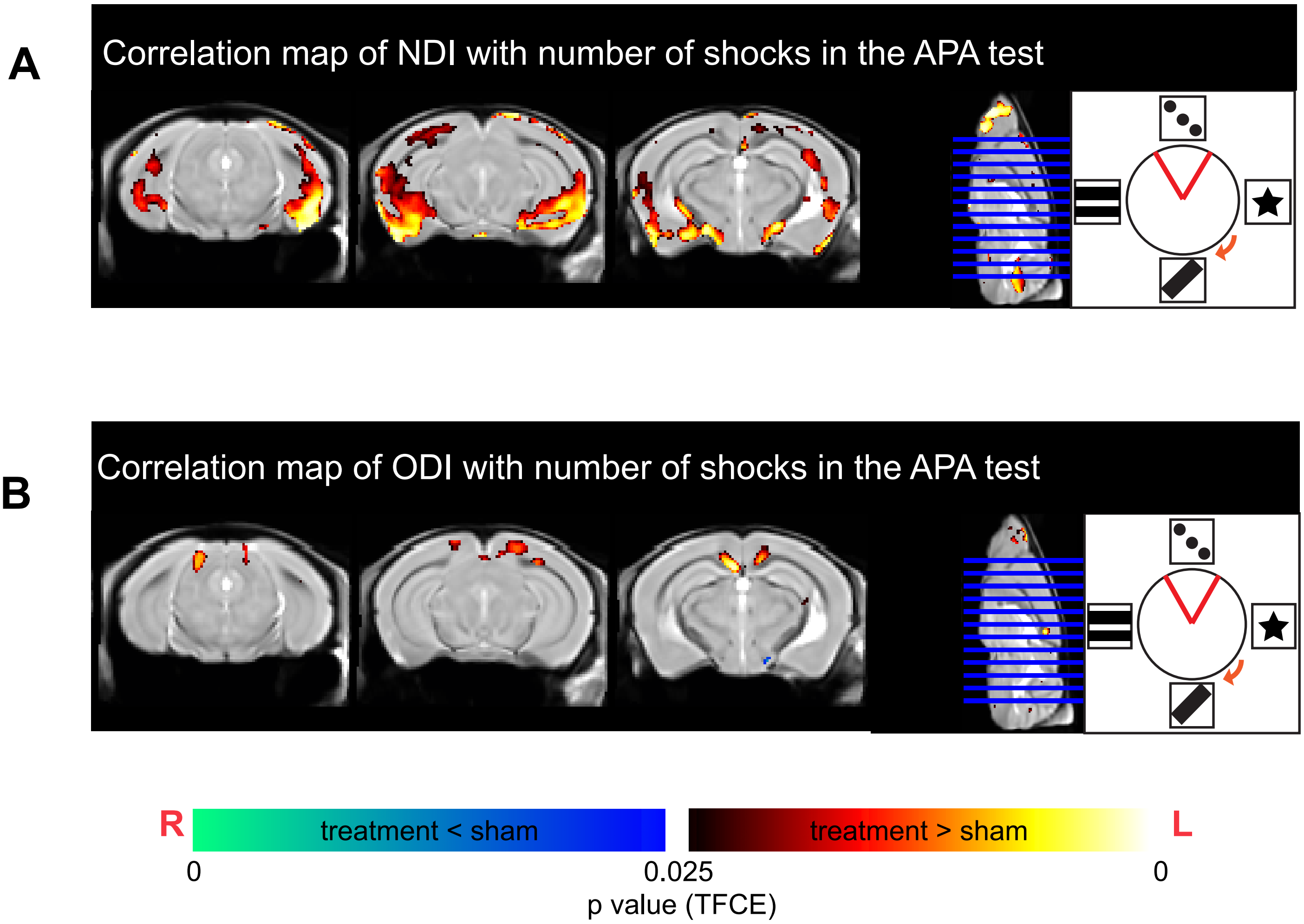
A
Correlation map of NDI with number of shocks in the APA test
B
Correlation map of ODI with number of shocks in the APA test
R
treatment < sham
treatment > sham
L
0
0.025
0
p value (TFCE)

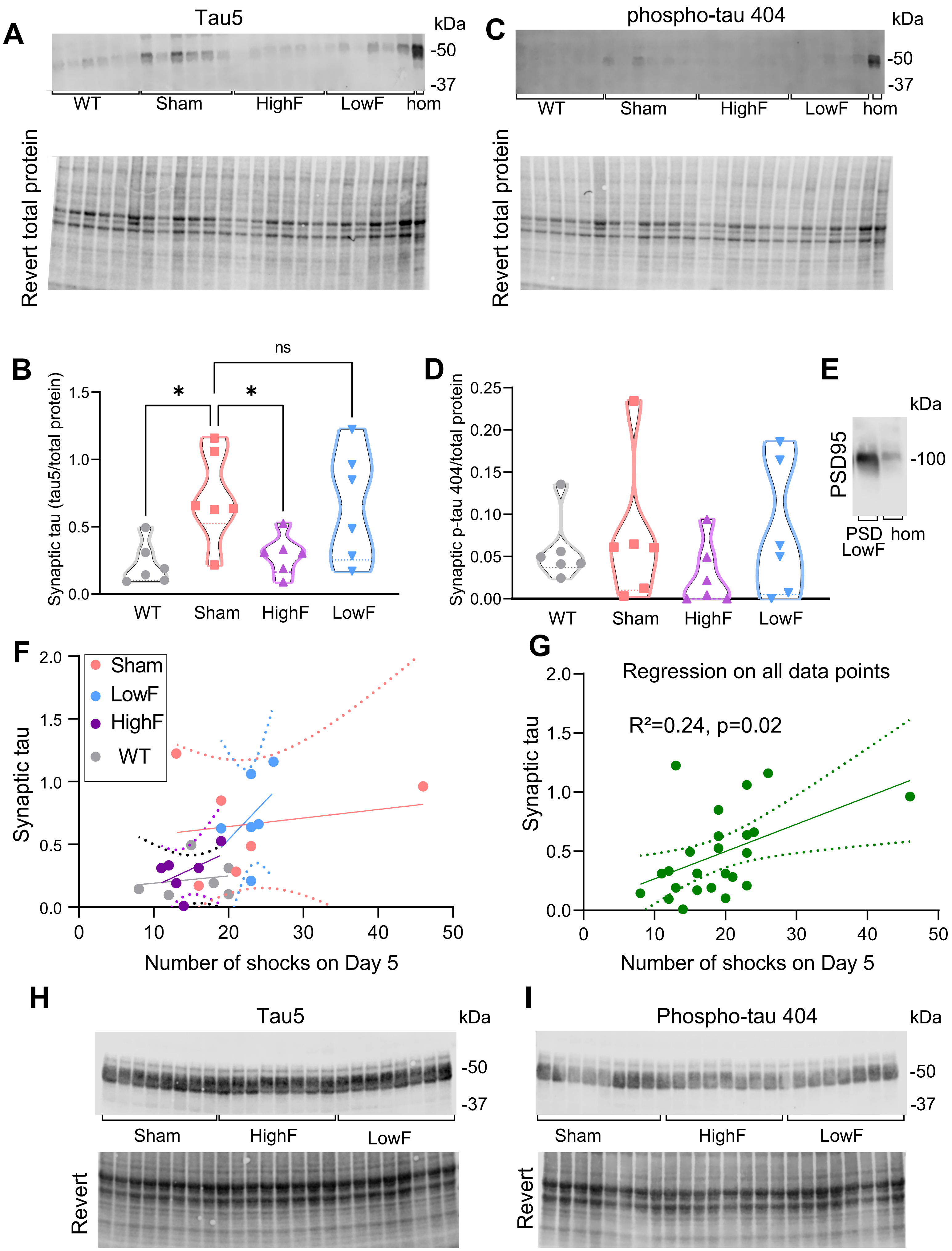

A
Tau5
kDa
-50
-37
WT
Sham
HighF
LowF
hom
Revert total protein
C
phospho-tau 404
kDa
-50
-37
WT
Sham
HighF
LowF
hom
Revert total protein
B
ns
*
*
Synaptic tau (tau5/total protein)
1.5
1.0
0.5
0.0
WT
Sham
HighF
LowF
D
Synaptic p-tau 404/total protein
0.25
0.20
0.15
0.10
0.05
0.00
WT
Sham
HighF
LowF
E
PSD95
kDa
-100
PSD LowF
hom
F
Sham
LowF
HighF
WT
Synaptic tau
2.0
1.5
1.0
0.5
0.0
0
10
20
30
40
50
Number of shocks on Day 5
G
Regression on all data points
R²=0.24, p=0.02
Synaptic tau
2.0
1.5
1.0
0.5
0.0
0
10
20
30
40
50
Number of shocks on Day 5
H
Tau5
kDa
-50
-37
Sham
HighF
LowF
Revert
I
Phospho-tau 404
kDa
-50
-37
Sham
HighF
LowF
Revert
